# Surgical interventions of isolated active mitral valve endocarditis: Predisposing factors and impact of neurological insults on final outcome

**DOI:** 10.1097/MD.0000000000010054

**Published:** 2018-03-16

**Authors:** Hsiu-An Lee, Chun-Yu Lin, Yung-Chang Chen, Shao-Wei Chen, Yu-Yun Nan, Kuo-Sheng Liu, Meng-Yu Wu, Yu-Sheng Chang, Jaw-Ji Chu, Pyng-Jing Lin, Feng-Chun Tsai

**Affiliations:** a Department of Thoracic and Cardiovascular Surgery; b Department of Nephrology, Chang-Gung Memorial Hospital, Linkou Medical Center, Chang Gung University, Taoyuan City, Taiwan.

**Keywords:** infective endocarditis, intracranial hemorrhage, mitral valve repair, neurologic complications

## Abstract

The feasibility and durability of mitral valve (MV) repair in active infective endocarditis (IE) has been reported, but proper management of perioperative neurological complications and surgical timing remains uncertain and may crucially affect the outcome.

In this single-center retrospective observational study, patients who underwent isolated MV surgery for active native IE in our institution between August 2005 and August 2015 were reviewed and analyzed. Patients who were operated on for healed IE or who required combined procedures were excluded from this study.

A total of 71 patients were enrolled in the study with a repair rate of 53.5% (n = 38). Isolated posterior leaflet lesion was found in 15 patients (21%) and was related to higher reparability (86.7%, *P* = .004). The overall in-hospital mortality was 10 (14.1%): 3 (7.9%) in the repair group and 7 (21.2%) in replacement group (*P* = .17). Prognosis was not related to age, preoperative renal function, cerebral emboli, or duration of antibiotics. The only significant predictor was postoperative intracranial hemorrhage (ICH) [odds ratio 14.628 (1.649–129.78), *P* = .04]. At a mean follow-up period of 43.1 months, neither recurrent endocarditis nor late cardiac death was observed in both groups.

Surgical timing and procedural options of MV surgery in active native IE did not make any difference, but occurrence of ICH after surgery jeopardized the final outcome. Routine preoperative brain imaging to detect silent ICH or mycotic aneurysm and aggressive treatment of these lesions may prevent catastrophe and optimize the results.

## 1 Introduction

In Taiwan, approximately 6.43 episodes of hospitalization for infective endocarditis (IE) per 100,000 people occur annually.^[1]^ Surgical intervention for active IE is highly challenging and carries high mortality and morbidity. Early referral to a first-rate heart center and proper management adhering to treatment guidelines are key factors for optimal outcomes, but the timing of surgery, especially in patients with neurological insults, and the choice of surgical procedure, either repair or replacement, remain controversial.^[2,3]^ Although mitral valve (MV) repair is recommended in preference to MV replacement,^[3]^ concerns persist regarding the feasibility of satisfactory repair in IE patients with extensive destruction of leaflets and subvalvular structures. Owing to the variations in the degree of structural destruction and the surgeon's familiarity with MV repair techniques, a variable MV reparability of 35% to 75% has been reported in active IE.^[4–7]^

Neurological complications, mainly related to embolism from vegetations, occur in 15% to 30% of IE patients,^[3]^ with ischemic stroke occurring in 14%, encephalopathy or meningitis in 6%, hemorrhages in 4%, and brain abscess in 1%.^[8]^ These neurological complications may jeopardize the surgical outcome and often are the main reason to delay the surgery. Current guidelines suggest that computed tomography (CT) or magnetic resonance imaging (MRI) should be performed in IE patients presenting with neurological symptoms but do not recommend routine performance of brain imaging in all IE patients, who might have silent intracranial lesions.^[3]^ Through the review of 10-year data of a single, tertiary hospital, we aim to identify the factors that influenced the reparability of MV in active IE, the proper timing for surgical intervention, and the impact of concomitant neurological events that occurred before and after surgery.

## 2 Materials and methods

This study was conducted after the approval of our institutional ethics committee (IRB reference No. 201600596B0). Because this was a retrospective study, the need for individual informed consent was waived. Data were obtained from the department's database and through a medical chart review. Patients who underwent isolated MV surgery for active native IE in our institution between August 2005 and August 2015 were identified. Patients who were operated on for healed IE, who required combined procedures, or who had missing data were excluded. Preoperative demographics, associated comorbidities, surgical procedures, perioperative recoveries, and midterm outcomes were collected and analyzed.

### 2.1 Definitions of isolated active IE

The diagnosis of IE was made according to modified Duke's criteria.^[9]^ Active IE was defined as the presence of vegetation or an abscess; evidence, such as fever and leukocytosis, of infections; or ongoing treatment with antibiotics.

### 2.2 Preoperative evaluation

All patients had received blood cultures and transthoracic echocardiography for definitive diagnosis, periodical follow-up, and in case of suspicious recurrent or uncontrolled local infection. In accordance with earlier guidelines, preoperative brain CT or MRI was to be performed only in patients who exhibited neurological symptoms.^[3]^ However, after the occurrence of some devastating postoperative neurological events, even in patients with no previous neurological symptoms, routine preoperative brain imaging was recommended for all patients, if conditions permitted.

### 2.3 Timing of surgery

Preoperative antibiotic treatment was administered for ≥7 days preferably to sterilize the patient. However, emergent surgery (within 24 hours) was performed regardless of preoperative antibiotic duration, if the severe valvular destruction resulted in refractory pulmonary edema or unstable hemodynamics requiring ventilator, inotropic, or vasopressor support. Urgent operation (within a few days) was arranged when any of the following conditions existed: aggravated heart failure symptoms, progression of sepsis, annular abscess, enlarging vegetation, recurrent systemic embolism despite appropriate antibiotic therapy, or large mobile vegetations.

**Figure 1:**
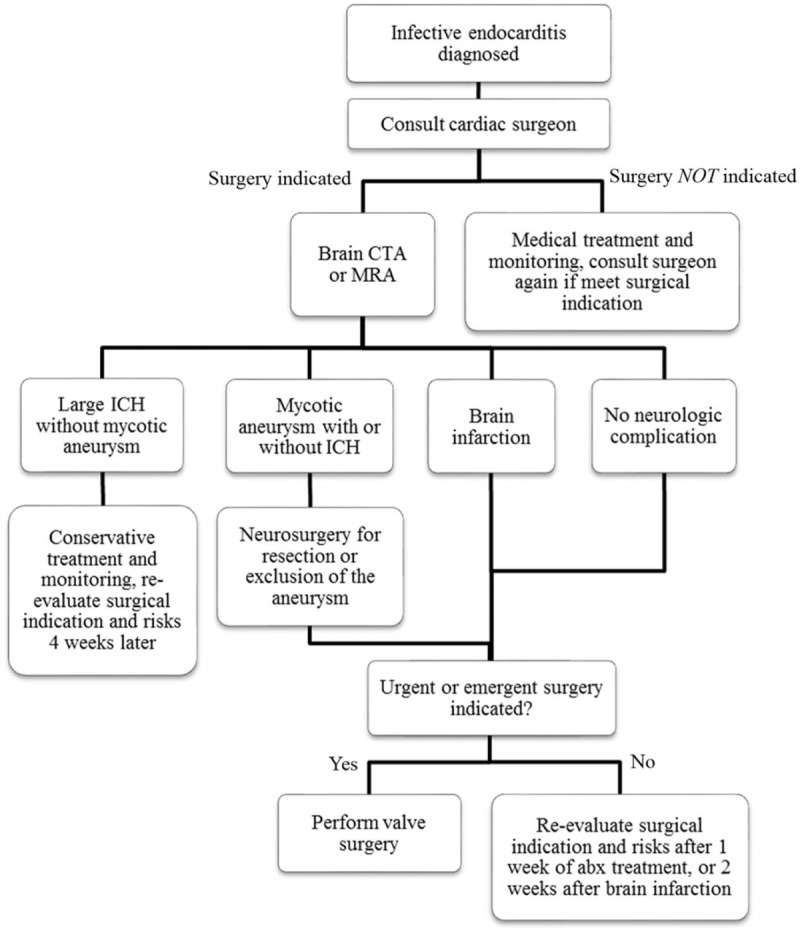
Flowchart of the survey and management of neurological complications in patients with active infective endocarditis. CTA = computed tomographic angiography, ICH = intracranial hemorrhage. MRA = magnetic resonance angiography.

Surgical intervention in patients with recent cerebrovascular events may increase the risk of postoperative intracranial hemorrhage (ICH). Therefore, valve surgery was liberally postponed for 2 weeks if brain infarction was present, as long as no urgent or emergent operative indications were noted. Patients with ICH were conservatively managed for ≥4 weeks, followed by reevaluation for surgical intervention. However, if a causative cerebral mycotic aneurysm of the ICH was identified and properly excised or excluded by the neurosurgeon, an urgent or emergent MV operation was still performed, if indicated (Fig. 1).

### 2.4 Surgical technique

Median sternotomy and cardiopulmonary bypass were set up in the normal fashion. Intraoperative transesophageal echocardiography was used to assess the patient's IE lesions and evaluate the patient's MV competency. The feasibility of MV repair was considered after complete debridement of the infective tissues, and the decision of repair or replacement was dependent on the surgeon's discretion with regard to the degree of valve destruction and patient characteristics such as age, heart function, comorbidity, and preoperative hemodynamics. The Carpentier principle for MV repair^[10]^ was used for all valves that were repaired. Artificial chordae were used in case of severe subvalvular destruction with no healthy secondary chordae. Ring annuloplasty was applied once the annulus was dilated to ensure the long-term durability. If replacement was necessary, the choice of prosthesis was based on the patient's preference after full explanation and discussion. Uninfected chordae were preserved for superior ventricular function reservation.

### 2.5 Statistical analysis

Statistical analyses were performed using SPSS for Windows (Version 22.0, SPSS, Chicago, IL). Data are presented as mean ± standard deviation for the numerical variables and as percentages for the categorical data. In all the analyses, statistical significance was set at *P* < .05. Univariate analyses were performed using the independent *t*-test, Mann–Whitney *U* test, χ^2^ test, or Fisher exact test to compare the clinical demographics and postoperative complications. Significant univariables for in-hospital mortality (*P* < .05) were dichotomized on the basis of the cutoff values, which were determined through receiver-operating characteristic (ROC) curve analysis. These dichotomized risk factors were tested through multivariate logistic regression analysis, the Hosmer–Lemeshow test, and area under ROC curve (AUROC) analysis to create a prediction model of in-hospital mortality. The Cox proportional hazards model was used to generate adjusted cumulative event-free survival curves that compared the MV repair and MV replacement groups. Parameters with *P* < .05 in the univariate Cox regression analysis were included in the multivariate Cox regression analysis.

## 3 Results

Between August 2005 and August 2015, 196 patients received open-heart surgeries for active IE in our hospital. MV involvement was noted in 143 patients (84.6%). Among these, 71 underwent isolated MV surgery for native valve IE, whereas MV repair and MV replacement were performed in 38 (53.5%) and 33 (46.5%) patients, respectively.

### 3.1 Preoperative demographics, comorbidities, and clinical findings

Table 1 lists the patient demographics, comorbidities, and preoperative characteristics. The mean age was 47.6 ± 18.2 years, and 27 patients were female (38%). Patients in the MV replacement group were older (*P* = .007), had a higher incidence of diabetes (*P* = .02), and a trend of larger vegetations (*P* = .72). Preoperative systemic embolic episodes, including 24 (33.8%) cerebral events, were documented in 34 patients (47.9%). Recurrent cerebral events and peripheral embolisms occurred in 5 and 2 patients, respectively. Severe mitral regurgitation (MR) was found in 53 patients, including 30 patients presenting with heart failure. Ten patients had uncontrolled sepsis, including 1 annular abscess. The size of the largest vegetations were <10 mm, 10.0 to 14.9 mm, 15.0 to 19.9 mm, 20 to 29.9 mm, and ≥30 mm in 19, 17, 17, 17, and 1 patient, respectively.

**Table 1:**
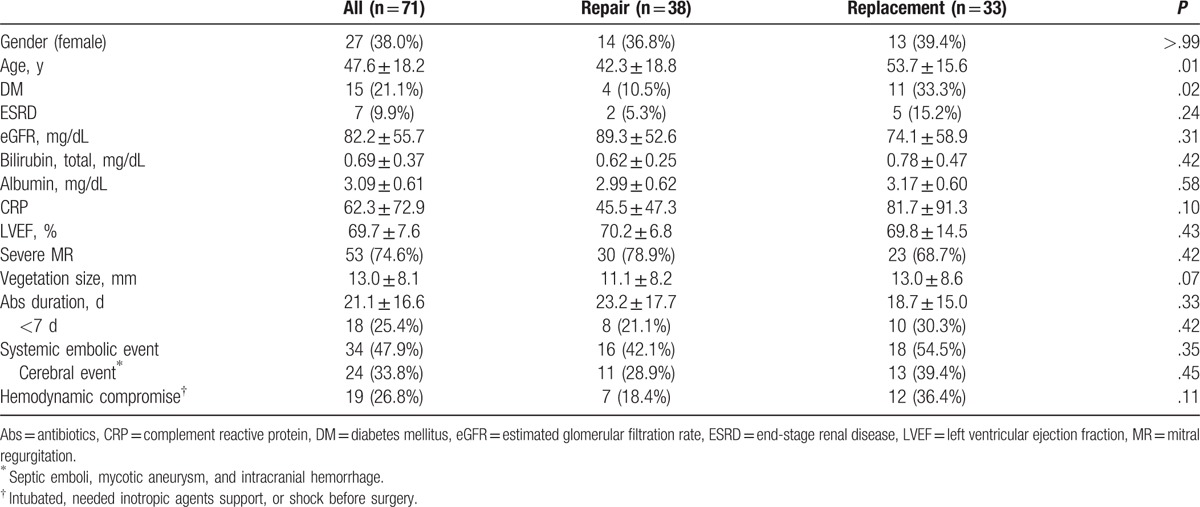
Preoperative clinical, laboratory, and echocardiography findings of patients who underwent isolated mitral valve surgery for active endocarditis.

Positive blood cultures were obtained in 52 (73.2%) patients, with the identification of Streptococcus and Staphylococcus species in 35 (49.3%) and 11 (15.5%) patients, respectively. Table 2 lists the details of various culture reports. We found no correlation between the infecting organisms and repairability. The mean duration of preoperative antibiotic treatment was 21.1 ± 16.6 days but 18 (25.4%) patients received antibiotic treatment for periods <7 days due to the necessity of emergent surgery.

**Table 2:**
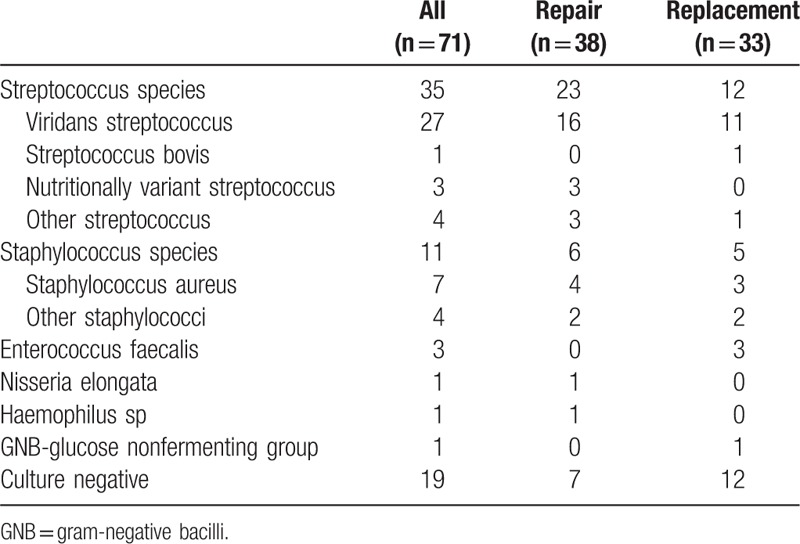
Microbiological findings of patients who underwent isolated mitral valve surgery for active endocarditis.

### 3.2 Surgical information

Table 3 lists the intraoperative findings. The majority of patients (66.2%, n = 47) had operations involving both leaflets, which contributed to the lowest possibility of MV repair (42.6%, *P* = .01). Factors associated with high reparability were isolated posterior leaflet lesions (86.7%, *P* = .004) and surgeries performed by senior surgeons (75.6%, *P* < .001). Among 38 MV repair cases, 81 repair procedures were performed; the average number of repair procedures per patient was 2.13. The repairs included 31 extensive resections, 7 pericardial patches, 16 chordae maneuvers, and 34 ring annuloplasties. Among 33 MV replacement cases, bioprostheses were implanted in 25 (75.8%) patients. Table 4 lists the details of the surgical procedures.

**Table 3:**
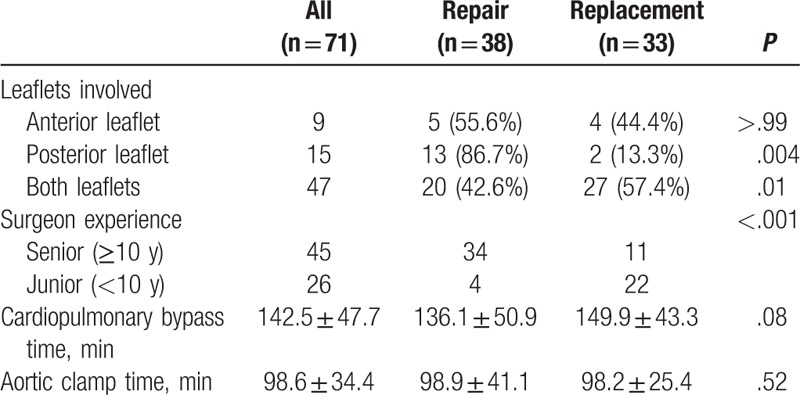
Leaflet involvement and surgical information of patients who underwent isolated mitral valve surgery for active endocarditis.

**Table 4:**
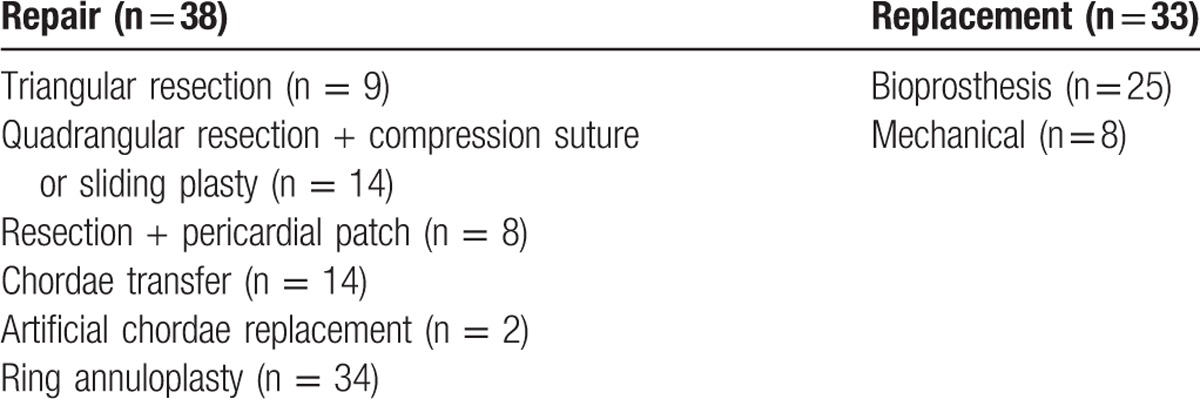
Surgical procedures for mitral valve repair and the types of prosthetic valves used in mitral valve replacement.

### 3.3 Risks of in-hospital mortality

The overall in-hospital mortality was 10 patients (14.1%), with 3 (7.9%) and 7 (21.2%) patients from the repair and replacement groups (*P* = .17), respectively. The causes of mortality were severe sepsis (n = 7), acute respiratory distress syndrome (n = 1), large ICH with cerebral herniation (n = 1), and sudden collapse in a patient with postoperative subdural hemorrhage and hemiparesis. Table 5 lists all the possible predictors of in-hospital mortality. Multivariate analysis showed that the occurrence of postoperative ICH [odds ratio 14.628 (1.649–129.78), *P* = .04] was the only significant predictive factor for in-hospital mortality.

**Table 5:**
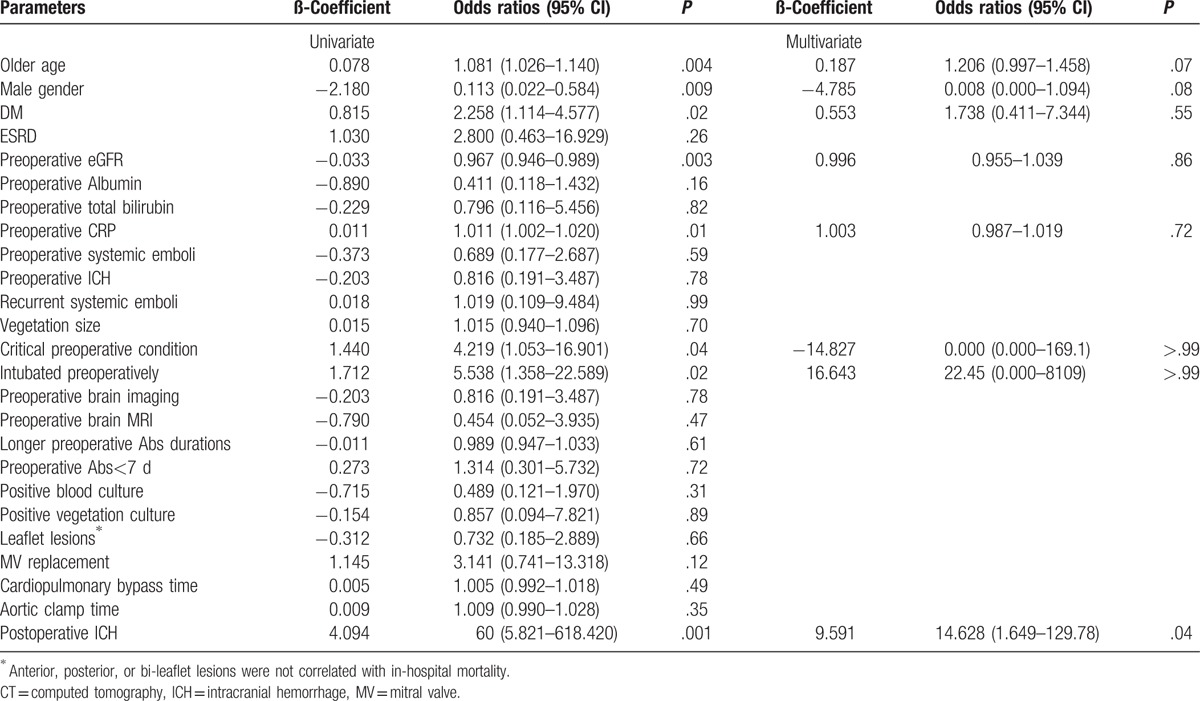
Logistic regression of the multivariate analysis for in-hospital mortality.

### 3.4 Brain imaging and cerebrovascular events

**Figure 2:**
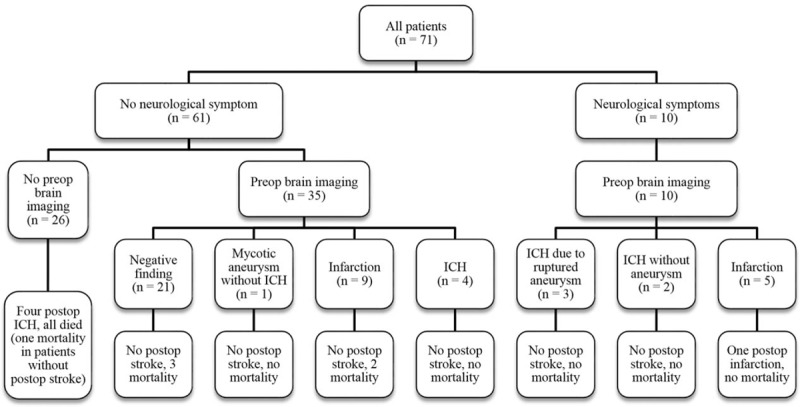
Flowchart describing various proportions of patients who had preoperative neurological complications, patients who received preoperative brain imaging, the imaging findings, patients who suffered from postoperative stroke, and in-hospital mortalities. postop = postoperative, preop = preoperative.

Figure 2 illustrates the numbers of patients with preoperative neurological symptoms, preoperative brain imaging, their imaging results, and hospital outcomes. Ten patients experienced preoperative neurological symptoms due to ICH or infarction. Three of them suffered from ruptured cerebral aneurysms; neurosurgeries for hematoma evacuation and aneurysm excision were performed, followed by successful valve surgeries. All 10 patients had no postoperative strokes and survived to discharge.

Of 61 patients who did not have preoperative neurological symptoms, 35 underwent brain imaging. Uncomplicated cerebral aneurysm was observed in 1 patient, who received transarterial embolization of the aneurysm before valve surgery, and was discharged smoothly. Cerebral infarction and ICH (without aneurysm) were observed in 9 and 4 patients, respectively; none of them had postoperative stroke. The other 26 patients did not receive any preoperative brain imaging. Postoperative ICH occurred in 4 of them, and none of those 4 patients survived to discharge. One died because of cerebral herniation due to large ICH, 2 had pneumonia and severe sepsis, possibly related to impaired cough function and mobility after ICH, and the other sudden collapsed, suspect due to airway obstruction.

### 3.5 Midterm outcome

**Figure 3:**
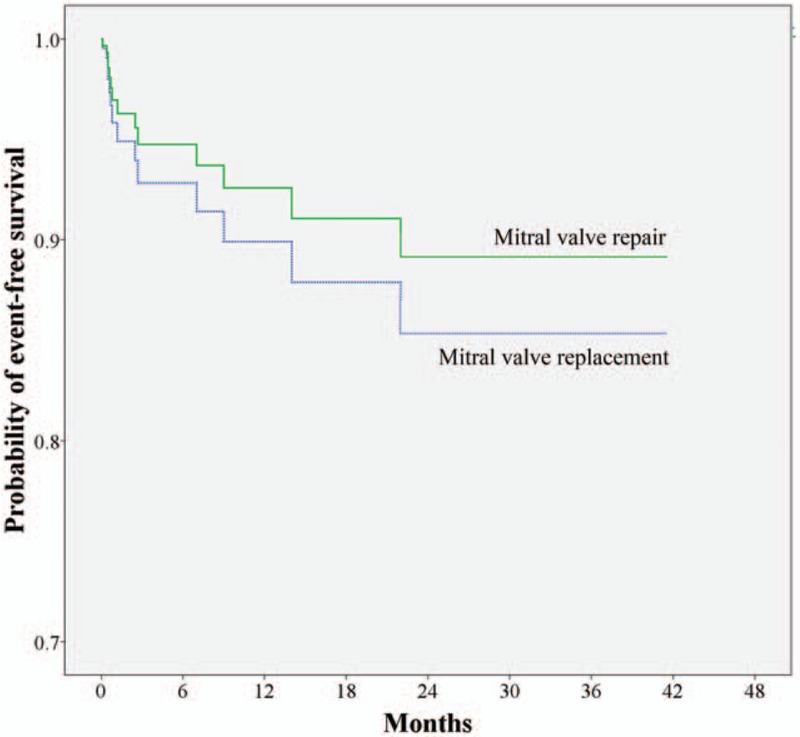
Adjusted survival curves were generated using the multivariate Cox proportional hazards model to compare the adjusted midterm event-free survival probability between the mitral valve repair and mitral valve replacement groups (*P* = .67).

Complete follow-up was achieved in 58 (95.1%) patients. At a mean follow-up of 43.1 months, 3 late mortalities occurred in the replacement group, but the causes of mortality were not relevant to cardiac events. Recurrent moderate or severe MR was noted in 3 patients in the repair group, one of whom underwent reoperation for MV replacement 14 months later. One patient in the replacement group experienced early bioprosthetic valve degeneration with significant mitral stenosis 4 years postoperatively. In both groups, recurrent endocarditis and late cerebrovascular events did not occur. Figure 3 illustrates that the adjusted survival curve showed no difference in the midterm event-free survival (free of recurrent endocarditis, cerebrovascular events, and reoperation) between the repair and replacement groups (*P* = .67).

## 4 Discussion

### 4.1 Repair or replacement

Theoretically, the outcomes of MV repair are superior to those of replacement in terms of long-term durability and thromboembolic complications. In IE patients, the benefit of MV repair over replacement has been demonstrated,^[4,11,12]^ but selection bias has not been eliminated in previous retrospective studies. Not all studies support MV repair; this is especially notable in series that included only active IE cases.^[5,6]^ Considering the variability in clinical status, extent of tissue destruction, and surgeon's experience, conducting prospective randomized clinical trials to compare these 2 therapeutic options may not be practical. In our study, both hospital and midterm survival in patients receiving replacement and repair were comparable, with no recurrent endocarditis in both groups; however, patients in the replacement group were significantly older and had more comorbidities. We do not advocate routine MV replacement in IE patients but believe that an adequate debridement and prompt replacement are justified in older patients and in patients with unstable hemodynamics or severely destructed valves, for which complex repair is expected. The complete excision of infected tissue is the golden rule for surgery in active IE and should never be compromised by the concern for the reparability of the valve.

### 4.2 Antibiotic duration

A major concern of valve surgery in active IE is the occurrence of recurrent endocarditis because of incomplete preoperative antibiotic treatment.^[13]^ In this study, 30 (42.3%) patients received antibiotic treatment for <14 days preoperatively; of those patients, 18 (25.4%) received treatment for periods <7 days. Shorter preoperative antibiotic duration did not jeopardize the surgical outcome or lead to recurrent endocarditis. Accordingly, whenever emergent or urgent surgery is indicated, preoperative antibiotic duration should never be a reason to postpone the operation.

### 4.3 Correlations of in-hospital mortality to preoperative risk factors

Early postoperative mortality in active native MV endocarditis has been reported to be between 3% and 17.1%, mainly depending on the preoperative status.^[4–7,14–16]^ In our study, 19 (26.8%) patients underwent emergent surgery, among which 10 (14.1%) were intubated and 1 needed an extracorporeal life support system preoperatively. Data analysis showed that in-hospital mortality was not related to any preoperative factors, including age, sex, diabetes, renal dysfunction, preoperative hemodynamics, preoperative neurological events, and surgical options. We may attribute this to timely surgical intervention that prevented irreversible organ damage. Early surgery, if indicated, is the key to success, especially when patients are experiencing heart failure, or uncontrolled sepsis, or both. A delay in intervention because of inappropriate expectations that conditions may be stabilized through medical treatment can result in multiple systemic organ failure and less optimal outcomes even if cardiac problems can be solved.

### 4.4 Prevention of cerebrovascular events

In this study, the only significant predictive factor for in-hospital mortality that we found was postoperative ICH. Postoperative ICH occurred in 4 of our patients, and all 4 of them died before discharge. None of these patients experienced neurological symptoms before operation, neither did they receive brain imaging preoperatively.

Neurological sequela secondary to IE, such as cerebral mycotic aneurysm, ICH, and embolic infarction, are not necessarily symptomatic, but they increase the risk of postoperative ICH, and can lead to disastrous results. Hence, we routinely perform preoperative brain CT or MRI for every IE patient after these catastrophic postoperative complications. In our study, asymptomatic cerebral mycotic aneurysm (n = 1) and silent ICH (n = 4) were identified and successfully managed without any sequela at discharge. To prevent postoperative ICH caused by silent intracranial lesions, we believe that preoperative brain CT or MRI should be a routine survey in active IE patients.

### 4.5 Limitations of the study

Despite the promising results of this study, several important limitations must be addressed. First, this was an observational study with a limited number of cases, suggesting the existence of bias regarding the nonhomogeneity between the 2 groups. Second, as a retrospective study, hemodynamic profiles, such as changes in central venous pressure, pulmonary artery or wedge pressure, cardiac output, and inotropic medication dosage, were not analyzed because of missing or incomplete records. This might hinder more detailed analyses of both pre- and postoperative hemodynamic fluctuation. Third, only 45 of our 71 patients received preoperative brain imaging, and the imaging modality also varied, affecting the complete evaluation of preoperative neurological complications. Finally, the decision of repair or replacement was made at the individual surgeon's discretion without consensus or reference to a protocol. The particular experience levels, confidence levels, and opinions of individual surgeons may provide widely different policies regarding treatment options.

## 5 Conclusions

In our study, neither surgical timing nor procedural options of MV surgery in active native IE made any difference to outcomes. Both MV repair and replacement can provide good durability and freedom from recurrent endocarditis, even without complete courses of antibiotics, as long as prompt intervention and complete debridement are performed. However, the occurrence of postoperative ICH significantly jeopardizes the outcome. Routine preoperative brain imaging to detect silent ICH or mycotic aneurysm, and aggressive treatment of these lesions are strongly recommended to prevent catastrophe and optimize the results.

## 6 Author contributions

**Conceptualization:** H.-A. Lee, C.-Y. Lin, J.-J. Chu, P.-J. Lin, F.-C. Tsai.

**Data curation:** H.-A. Lee, C.-Y. Lin.

**Formal analysis:** H.-A. Lee, Y.-C. Chen, M.-Y. Wu.

**Investigation:** S.-W. Chen.

**Methodology:** Y.-C. Chen, M.-Y. Wu.

**Project administration:** F.-C. Tsai

**Resources:** Y.-Y. Nan, K.-S. Liu, Y.-S. Chang, J.-J. Chu, P.-J. Lin.

**Supervision:** C.-Y. Lin, S.-W. Chen, Y.-Y. Nan, K.-S. Liu, M.-Y. Wu, Y.-S. Chang, F.-C. Tsai.

**Writing – original draft:** H.-A. Lee.

**Writing – review & editing:** S.-W. Chen, F.-C. Tsai.

## References

[1] Shih CJ Chu H Chao PW Long-term clinical outcome of major adverse cardiac events in survivors of infective endocarditis: a nationwide population-based study. Circulation 2014;130:1684–91. 25223982 10.1161/CIRCULATIONAHA.114.012717

[2] Thuny F Grisoli D Collart F Management of infective endocarditis: challenges and perspectives. Lancet 2012;379:965–75. 22317840 10.1016/S0140-6736(11)60755-1

[3] Habib G Lancellotti P Antunes MJ 2015 ESC Guidelines for the management of infective endocarditis: the Task Force for the Management of Infective Endocarditis of the European Society of Cardiology (ESC). Endorsed by: European Association for Cardio-Thoracic Surgery (EACTS), the European Association of Nuclear Medicine (EANM). Eur Heart J 2015;36:3075–128. 26320109 10.1093/eurheartj/ehv319

[4] Ruttmann E Legit C Poelzl G Mitral valve repair provides improved outcome over replacement in active infective endocarditis. J Thorac Cardiovasc Surg 2005;130:765–71. 16153926 10.1016/j.jtcvs.2005.03.016

[5] de Kerchove L Vanoverschelde JL Poncelet A Reconstructive surgery in active mitral valve endocarditis: feasibility, safety and durability. Eur J Cardiothorac Surg 2007;31:592–9. 17270457 10.1016/j.ejcts.2007.01.002

[6] Jung SH Je HG Choo SJ Surgical results of active infective native mitral valve endocarditis: repair versus replacement. Eur J Cardiothorac Surg 2011;40:834–9. 21459600 10.1016/j.ejcts.2011.01.016

[7] Ragnarsson S Sjogren J Stagmo M Clinical presentation of native mitral valve infective endocarditis determines long-term outcome after surgery. J Card Surg 2015;30:669–76. 26123359 10.1111/jocs.12591

[8] Garcia-Cabrera E Fernandez-Hidalgo N Almirante B Neurological complications of infective endocarditis: risk factors, outcome, and impact of cardiac surgery: a multicenter observational study. Circulation 2013;127:2272–84. 23648777 10.1161/CIRCULATIONAHA.112.000813

[9] Li JS Sexton DJ Mick N Proposed modifications to the Duke criteria for the diagnosis of infective endocarditis. Clin Infect Dis 2000;30:633–8. 10770721 10.1086/313753

[10] Carpentier A Adams DH Filsoufi F Carpentier's Reonstructive Valve Surgery. Philadelphia, PA: Saunders Elsevier; 2010.

[11] Feringa HH Shaw LJ Poldermans D Mitral valve repair and replacement in endocarditis: a systematic review of literature. Ann Thorac Surg 2007;83:564–70. 17257988 10.1016/j.athoracsur.2006.09.023

[12] Shang E Forrest GN Chizmar T Mitral valve infective endocarditis: benefit of early operation and aggressive use of repair. Ann Thorac Surg 2009;87:1728–33. 19463586 10.1016/j.athoracsur.2009.02.098

[13] Thuny F Beurtheret S Mancini J The timing of surgery influences mortality and morbidity in adults with severe complicated infective endocarditis: a propensity analysis. Eur Heart J 2011;32:2027–33. 19329497 10.1093/eurheartj/ehp089

[14] Zegdi R Debieche M Latremouille C Long-term results of mitral valve repair in active endocarditis. Circulation 2005;111:2532–6. 15867185 10.1161/01.CIR.0000165122.08660.1A

[15] de Kerchove L Price J Tamer S Extending the scope of mitral valve repair in active endocarditis. J Thorac Cardiovasc Surg 2012;143 (4 Suppl.):S91–5. 22306214 10.1016/j.jtcvs.2012.01.049

[16] Muehrcke DD Cosgrove DM 3rd Lytle BW Is there an advantage to repairing infected mitral valves? Ann Thorac Surg 1997;63:1718–24. 9205173 10.1016/s0003-4975(97)00271-3

